# Hesitancy Over the COVID-19 Vaccine Among Various Healthcare Workers: An International Narrative Review

**DOI:** 10.7759/cureus.53059

**Published:** 2024-01-27

**Authors:** Hussain A Aldakhlan, Abdul S Khan, Donna Alabdulbaqi

**Affiliations:** 1 Medicine, Saudi Board of Preventive Medicine, Alahsa, SAU; 2 Familly Medicine, King Faisal University, Alhsa, SAU; 3 Medicine, Saudi Health Council, Riyadh, SAU

**Keywords:** vaccine, resistant, hesitancy, healthcare workers, covid-19

## Abstract

Healthcare workers (HCWs) are role models in their communities. If they receive the COVID-19 vaccine, many people are likely to follow and have the vaccine. If HCWs are hesitant or resistant to taking the vaccine, this may impede the efforts to implement the vaccine, reach herd immunity, and eliminate the pandemic. In this narrative review, we reviewed previous studies on hesitancy over COVID-19 vaccination among different healthcare professions and people in the medical field, such as primary HCWs, dentists, nurses, and medical students. We reviewed the common reasons and associated factors for hesitancy toward the COVID-19 vaccine among different healthcare professions. The following keywords were used in the database search: COVID-19 AND vaccine AND hesitancy AND healthcare workers. We searched for articles using the PubMed, Scopus, and Google Scholar databases. We found HCW professions with various rates of hesitancy, including primary healthcare center (PHC) workers (50%), medical students (45%), nurses (21%), and dentists (18%). Hesitancy toward booster doses was also found in HCWs who had taken primary doses (2.8% to 26%). Race and ethnicity also influenced hesitancy rates, with Black individuals being the most hesitant group. The most common reasons were concerns about the safety and adverse effects of the vaccine, insufficient information, and a lack of confidence in healthcare policies. Despite varying rates of HCW hesitancy after the vaccine's release, this hesitancy is expected to negatively affect efforts to achieve widespread vaccination. The recommendations to policymakers to address these concerns are raising the awareness of PHC doctors because they are the easiest to reach and are the first line for patient information, improving communication with the HCWs through all channels (e.g., webinars, e-mails, and social media), and inviting HCWs to online meetings or workshops with the healthcare policymakers so the policymakers can listen to their concerns and recommendations. Correctly addressing the issue of HCWs' vaccination hesitancy can support efforts to contain the pandemic.

## Introduction and background

In December 2019, the Chinese Center for Disease Control and Prevention detected a new virus in Wuhan City, with clinical symptoms ranging from asymptomatic to severe [1]. The virus spread quickly, and the World Health Organization (WHO) declared it a pandemic, warning the world about its transmission and prevention methods [2]. The pandemic initially overwhelmed public health measures in place and threatened to severely burden health systems and economies globally. Developing an effective vaccine became imperative to protect populations and economies. On March 3, 2020, the first clinical trial of the COVID-19 vaccine was conducted in the United States. On September 11, 2020, the trial advanced to Phases 2 and 3, and eight vaccines were approved for early or limited use [3]. Many studies were carried out before the approval of these vaccines in European countries, and some people were not sure about taking the new COVID-19 vaccine [3] - they were vaccine-hesitant. Others did not want and were against vaccination against COVID-19. They were vaccine-resistant [3].

According to the WHO, vaccine hesitancy was among the top 10 threats to world health in 2019, along with climate change and air pollution, no coverage of primary healthcare services, the global influenza pandemic and noncommunicable diseases, weak and vulnerable settings, antimicrobial resistance, Ebola, and other high-threat pathogens. The term "vaccine hesitancy" refers to a hesitancy or refusal to receive a vaccine despite its availability. This factor impedes efforts to control the diseases that can be prevented by vaccination [4]. Millions of deaths have been prevented by vaccination. Vaccines are one of the most cost-effective ways to prevent disease. The reasons people prefer not to be vaccinated are complicated [4].

The vaccine advisory group to the WHO constructed the three-C model for vaccine hesitancy and its complacency. The model includes convenience or inconvenience, confidence or lack of confidence [4]. Extensive media campaigns featuring actors and celebrities were launched in many countries to promote vaccination among the general public. The influence of healthcare workers (HCWs) on vaccine hesitancy was less significant, given that they were required to get vaccinated to continue their professional duties in numerous nations. HCWs must be supported to provide accurate, credible information about the vaccine in their communities [4]. All HCWs should know and understand relevant definitions, such as "complacency", which is the perceived risk of diseases; "inconvenience", which is difficulty in accessing vaccines or reaching the healthcare site that provides the vaccine services; and "lack of confidence": the inability to have faith in the efficacy and safety of the vaccine and the healthcare system that administers it [5]. HCWs play a crucial role as primary defenders in the battle against the ongoing pandemic, given their heightened vulnerability to viral exposure [5]. Prioritizing the vaccination of HCWs becomes imperative. As community role models, healthcare workers significantly influence public perceptions and decisions regarding vaccines. The vaccination choices of healthcare workers serve as influential factors, inspiring a considerable number of individuals to follow suit. If the HCWs are hesitant or resistant to taking the vaccine, this may impede efforts to implement the vaccine and reach herd immunity to eliminate the pandemic [6]. There is little data about hesitancy or refusal of the COVID-19 vaccine among various HCWs [6]. This narrative review seeks to assess vaccine hesitancy and resistance within the healthcare workforce, focusing on various professions, including doctors, nurses, dentists, and others. The primary objectives are to determine the rate of COVID-19 vaccine hesitancy among different healthcare professions and to identify common reasons and associated factors contributing to this hesitancy. By addressing these questions, the review aims to provide valuable insights into the extent of vaccine hesitancy and to understand the underlying factors, thus contributing to the development of targeted strategies to address this crucial public health issue.

## Review

Materials and methods

**Figure 1:**
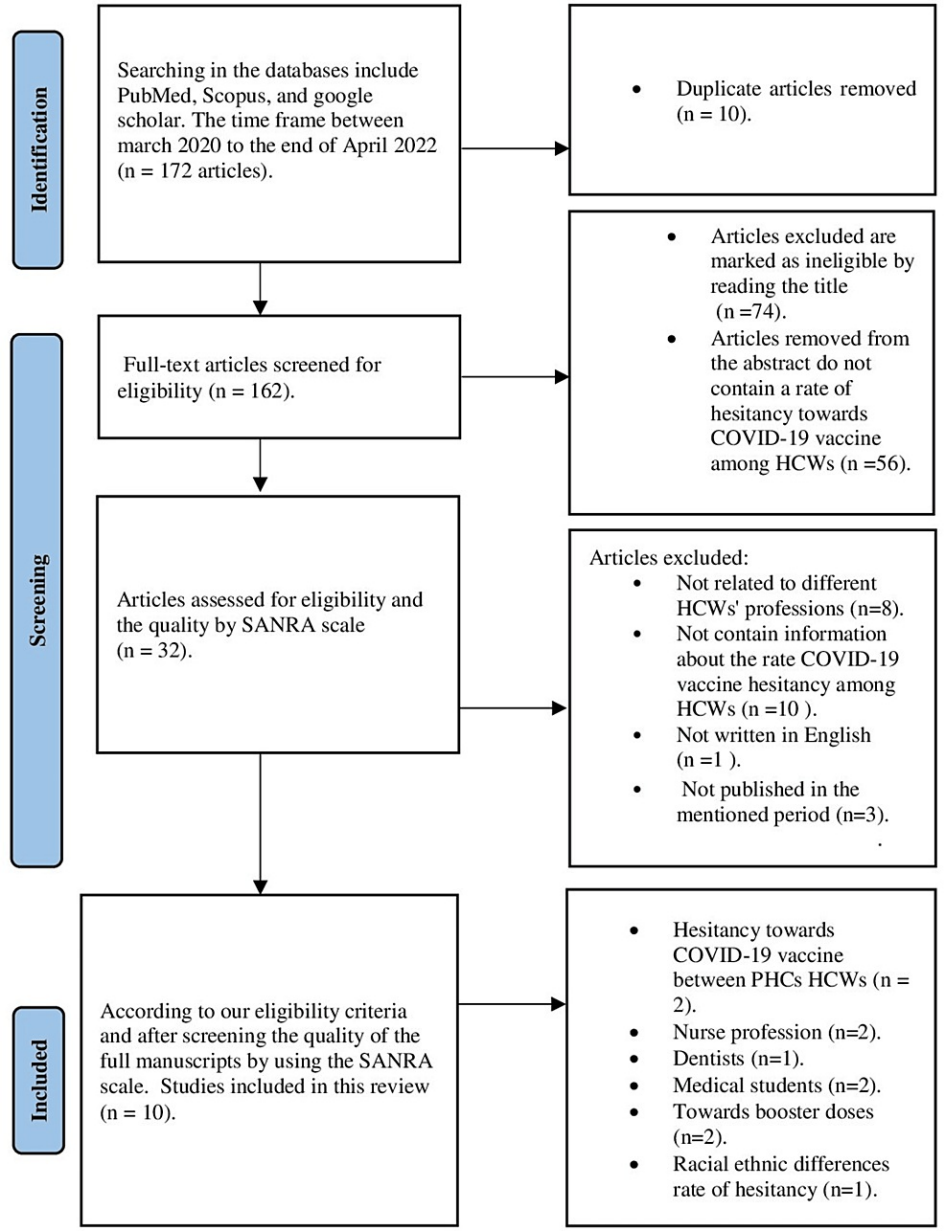
Flowchart of selection of the studies for review SANRA - Scale for the Assessment of Narrative Review Articles; HCWs - healthcare workers; PHC - primary healthcare

In this narrative review, we reviewed the literature to gather evidence on COVID-19 vaccine hesitancy among HCWs. The eligibility criteria for inclusion in this review were that the study must be related to COVID-19 vaccine hesitancy among HCWs and include all or most professions in the health system, such as doctors, nurses, dental and oral health professionals, medical technicians, health assistants, social workers, anyone working in health administration, or any people in the medical field, such as medical students. All study designs were included, such as randomized controlled trials, cohort, case-control, surveys, cross-sectional, and other designs. Any sample size was acceptable. The participants should all be HCWs, both male and female. We searched for articles using PubMed, Scopus, and Google Scholar. The articles had to be published from March 2020 to the end of April 2022. This timeframe was from starting the first dose of the COVID-19 vaccine until people completed the second or third dose in most countries. The following keywords were used in the database search: COVID-19 AND vaccine AND hesitancy AND healthcare workers. The titles and abstracts of the articles were screened for the eligibility criteria. The full manuscripts of the included articles were then screened using the Scale for the Assessment of Narrative Review Articles (SANRA) [7]. We excluded all studies that were not written in English, not related to hesitancy over the COVID-19 vaccine, and not related to HCWs' professions or people in the medical field. We excluded any research that did not provide information about the rate of COVID-19 vaccine hesitancy, its associated factors, and common reasons for hesitancy among HCWs, as clarified in Figure 1. By applying all these eligibility criteria, we selected those studies included in Table 1. To answer the review questions: What is the rate of hesitancy toward the COVID-19 vaccine among different HCWs' professions, and what are the common reasons and associated factors for this hesitancy.

Results

After applying our eligibility criteria, we included 10 studies comprising one scoping review and nine cross-sectional studies. Table 1 summarizes these studies.

**Table 1:** Summary of the included studies HCWs - healthcare workers

Title	Authors	Sample size	Period and site	Study design	Hesitancy rate toward the COVID-19 vaccine	Reason/associated factors
COVID-19 vaccine hesitancy and related factors among primary HCWs in a district of Istanbul.	İkiışık et al. [8]	323 participants	From December 25, 2020, to December 29, 2020, in a district of Istanbul	A cross-sectional study	Of 323, 139 of the participants were hesitant to receive the COVID-19 vaccine in this study.	Non-physician HCWs, women, and those aged less than 40 were less likely to accept the COVID-19 vaccine.
COVID-19 vaccine acceptance and hesitancy among primary HCWs in Singapore.	Koh et al. [9]	528 participants	From May to June 2021, in Singapore	A cross-sectional survey	Among 528 respondents, the vaccine acceptance rate was (94.9%) (n=501). Most of the participants have accepted the vaccine only (5%) were hesitant to receive the COVID-19 vaccine.	The top reasons for vaccine acceptance were exposure to COVID-19, a suspected or confirmed case, to protect family and friends, and to protect themselves because of high-risk jobs.
Beliefs and barriers associated with COVID-19 vaccine among Egyptian medical students.	Saied et al. [10]	2133 participants	During January 2021, Tanta and Kafrelsheikh Universities, two central public universities in the Delta region	A cross-sectional study	The hesitant group numbered 974 from 2133 (45%) participants.	Concerns regarding the vaccine's adverse effects were greater than concerns about the ineffectiveness of the vaccine and insufficient information regarding the vaccine itself.
COVID-19 vaccine hesitancy among medical students in India.	Jain et al. [11]	1068 participants	Five weeks from February 2 to March 7, 2021 in India	A cross-sectional study	Vaccine hesitancy was found among 113 students (10.6%).	Lack of awareness regarding vaccination eligibility, concern regarding adverse events and efficacy of the vaccine, and lack of trust in government were independently predictive of vaccine hesitancy.
COVID-19 vaccine refusal among nurses worldwide: review of trends and predictors.	Khubchandani et al. [12]	41,098 participants	From March 2020 to May 2021	A scoping review	The pooled prevalence rate of COVID-19 vaccine refusal was 20.7%.	Concerns about vaccine safety, side effects, and efficacy, misinformation, and lack of knowledge.
Vaccine hesitancy and uptake among nursing staff during an active vaccine rollout.	Baniak et al. [13]	276 participants	During February 2021 among nursing staff working in a large medical center in the central United States	A cross-sectional study	The total number of people hesitant over the COVID-19 vaccine was 16.3%.	Less than 10 years' work experience, concerns about long-term side effects, and the vaccine does not prevent people from having a second infection caused by the COVID-19 virus.
COVID-19 vaccine hesitancy and reasons for or against adherence among dentists.	Belingheri et al. [14]	761 participants	Italy, from December 23, 2020, through January 2, 2021	A cross-sectional study	The hesitancy rate toward the COVID-19 vaccine among dentists in this study was 18%.	The main reason for opposing vaccination was the lack of information.
COVID-19 vaccine booster doses hesitancy among HCWs in Singapore.	Koh et al. [15]	891 participants	In Singapore between January 1 and December 10, 2021	A retrospective cross-sectional study	Of the eligible participants for booster doses, 26% were hesitant.	HCWs hesitant to receive the first dose were 3.6 times more hesitant toward booster doses of the COVID-19 vaccine.
COVID-19 vaccine hesitancy and attitude toward booster doses among US HCWs.	Pal et al. [16]	1358 participants	In the United States. Data were collected between February 1, 2021 and March 31, 2021	A cross-sectional survey	Full data were available from 107 participants (7.9%) who were hesitant to receive either the first or second dose. Only three respondents (2.8%) were hesitant to take the second dose after receiving the first dose.	The most common reason for hesitancy toward COVID-19 vaccine booster doses among HCWs in the United States was getting side effects from the first dose.
Racial and ethnic differences in COVID-19 vaccine hesitancy among HCWs in two large academic hospitals.	Momplaisir et al. [17]	10,871 participants	From two academic hospitals in the United States over a three-week period in November and December 2020	A cross-sectional survey	COVID-19 vaccine hesitancy was highest among Black participants, compared to White and Asian individuals. Of 882 individuals, 732 were hesitant over the COVID-19 vaccine (83%).	This study found that COVID-19 vaccine hesitancy was extremely high among Black, Hispanic, Latino, and Asian HCWs compared with White HCWs.

Discussion

Previous Studies Exploring the Hesitancy Rate for the COVID-19 Vaccine Among HCWs

Due to the paucity of information regarding the COVID-19 vaccination among HCWs, a systematic review and meta-analysis were conducted to ascertain the extent and nature of vaccine hesitancy among HCWs. The systematic review included estimations from 35 international studies of published evidence on COVID-19 vaccine hesitancy among HCWs, with study sample sizes ranging from 123 to 16,158 participants, almost 2185 participants per study [18]. The prevalence of COVID-19 vaccination hesitancy worldwide in HCWs ranged from 4.3% to 72% [18]. The review reported the most common factors associated with an increase in hesitancy over the COVID-19 vaccine among HCWs who experienced side effects from this new vaccine. The previous studies provided evidence regarding the factors linked with COVID-19 vaccine acceptance, such as male gender, older age, and education level of HCWs, such as doctoral degree holders [18].

Previous Studies Exploring Common Reasons for COVID-19 Vaccine Hesitancy Among HCWs

On December 4, 2020, at the beginning of the COVID-19 vaccination campaign, a survey was carried out to examine the trends in HCWs' intentions to receive a COVID-19 vaccine and reasons for hesitancy. A total of 16,292 employees completed the survey. When asked if they would have the COVID-19 vaccine when it became available, 9015 respondents said yes (55%), 2658 employees said no (16.3%), and 4619 employees (28.4%) were undecided [19]. The most common reason for hesitancy among participants who responded no or who were undecided (6569) was concerns about the unknown risks of the vaccine (90.3%). More than half (57.4%) of 4187 employees cited concerns about known adverse effects, such as headaches and fatigue. In addition, 3226 employees (44.3%) decided not to vaccinate until other people had experience with this new vaccine and the availability of more information, and (21.1%) 1539 employees decided that they did not trust the promotion from the authority (Food and Drug Administration) process to vaccinate with this new vaccine [19].

Another study on common reasons for hesitancy was conducted in Pakistan. There was a serious threat of COVID-19 vaccine hesitancy, followed by failure to vaccinate and failure to reach herd immunity because of the spread of conspiracy theories through social media [20]. Thus, Pakistan was at risk of a COVID-19 vaccine failure because of the same previous experience of failure against the polio vaccine. Two political factors have appeared and spread in Pakistan: first, conspiracy theories against the COVID-19 vaccine; and second, the notion that the COVID-19 disease is a grand illusion and a conspiracy against Muslim countries. These notions have supported COVID-19 vaccine hesitancy among Pakistani people [20].

Transition to This Narrative Review Findings:

Transitioning to our narrative review findings, we investigated COVID-19 vaccine hesitancy rates among various HCWs professions and explored associated factors and common reasons. Specifically, we focused on:

Hesitancy Over the COVID-19 Vaccine Among Primary Healthcare (PHC) Doctors and Their Role

Family physicians in PHC are considered a trusted source of information about vaccines because of their distinct situation in the healthcare system and because they are the first line to face people in the community [8]. All people, whether they live in rural or urban regions, have easy access to primary healthcare services. The HCWs in PHC have a vital role in reducing all types of vaccine hesitancy in their communities. They can establish trust in the vaccine and advise people about the importance of vaccinations to protect themselves from many diseases. If a percentage of HCWs are hesitant to vaccinate, there is a problem for the healthcare system [8].

A cross-sectional study was carried out in Üsküdar, one of the districts in Istanbul, Turkey. The study ascertained the intentions and predictive factors for family physicians and family healthcare staff working in primary care centers regarding the COVID-19 vaccination. An online questionnaire was used to gather data from the family physicians and family HCWs between December 25 and 29, 2020. The participants were 323 HCWs from 44 family healthcare centers in the district [8]. The response number was 276 participants. From the professions, 126 physicians and 150 nurses and midwives participated. The results showed that 139 participants (50.4%) would take the vaccine, 80 participants (29%) were undecided, and 57 participants (20.7%) refused the vaccine. Half of the participants in this study were hesitant to accept the COVID-19 vaccine [8]. Non-physician HCWs, women, and those aged less than 40 years were the common associated factors for COVID-19 vaccine hesitancy in this study. These individuals were less likely to accept the COVID-19 vaccine. Individuals who were male, over 40 years old, working as physicians, and who previously received a seasonal influenza vaccination were linked with vaccination acceptance [8].

An additional study conducted in Singapore investigated COVID-19 vaccine hesitancy among HCWs in PHC. This study ascertained factors associated with COVID-19 vaccine hesitancy or acceptance among healthcare staff in primary centers. Between May and June 2020, an online cross-sectional survey was sent to staff in six PHC facilities. The distinct feature of this study was that it was carried out after the completion of the staff vaccination exercise. A total of 528 participants completed the survey questionnaire. From this total, 501 participants (94.9%) accepted, and only 5% were hesitant to take the COVID-19 vaccine [9]. According to this study, the three main reasons for vaccine acceptance were exposure to COVID-19 either as a suspected or confirmed case, safeguarding their family and friends, and safeguarding themselves because of their high-risk occupation as HCWs. There were no statistically significant differences relating to age, gender, profession, number of years working in healthcare settings, or previous influenza vaccination [9].

Hesitancy Over the COVID-19 Vaccination Among Medical Students

It is important to know the rate of COVID-19 vaccine hesitancy among medical students because they are the future healthcare providers. Additionally, it is important to know the factors associated with COVID-19 vaccine hesitancy and acceptance among medical students [10]. A cross-sectional study of the beliefs and barriers associated with the COVID-19 vaccine among Egyptian medical students in two central public universities, Tanta and Kafrealshaikh, carried out in January 2021, had 2133 participants. This study found that the hesitant group comprised 974 of the 2133 total participants (45%) [10]. The reasons for hesitancy among the Egyptian medical students was that most had concerns regarding the vaccine's adverse effects (96%), the ineffectiveness of the vaccine (93%), and insufficient information regarding the vaccine (72%). Hesitancy caused by unknown reasons was over 56% [10].

In India, in 2021, a similar cross-sectional study was carried out with a group of medical students. A total of 1068 students from 22 provinces in India participated in the online survey. Vaccine hesitancy was detected among 113 students (10.6%). COVID-19 vaccine hesitancy was detected in one out of every 10 medical students [11]. Common reasons for COVID-19 vaccine hesitancy among medical students in India were no information regarding vaccine competency, concerns regarding the side effects and safety of the vaccine, and no trust in the government [11].

Hesitancy Over the COVID-19 Vaccine Among HCWs in the Nursing Profession

Nurses play a significant role in providing long-term care and follow-up for COVID-19 patients. COVID-19 caused infection and death in a high number of nurses across the world [12]. To gather information on nurses' COVID-19 vaccination refusal and hesitancy rates, a scoping review was carried out. The review included 51 studies from 36 countries between March 2020 and May 2021, with a total sample size of 41,098 nurses. The pooled prevalence rate of COVID-19 vaccine hesitancy was nearly 21% [12]. The most common reasons for COVID-19 vaccine hesitancy were doubts about vaccine efficacy, safety and side effects, and mistrust of the authorities' sources of information on this new vaccine. The main factors contributing to vaccine acceptance were previous flu vaccination, male gender, and older age [12].

A cross-sectional study was carried out in the United States to assess the COVID-19 vaccine hesitancy and uptake among nursing staff during the active vaccine rollout. In February 2021, for nursing staff working in large medical centers in the United States, out of 276 participants, only (12%) expressed hesitancy at receiving the COVID-19 vaccine. The majority of the participants (82%) agreed to receive the COVID-19 vaccine, with only (6%) disagreeing. The total number of people hesitant to receive the vaccine was nearly 16% [13]. The most common reasons among hesitant nurses in this study were fewer years of work experience (less than 10 years), concern about long-term side effects, and that the vaccine does not prevent people from contracting a second infection caused by the COVID-19 virus. This study found no differences regarding age, sex, race, employment status, or marital status [13].

Hesitancy Over the COVID-19 Vaccine Among HCWs in the Dentistry Profession

COVID-19 spread significantly among HCWs. Dentists and oral HCWs have an increased risk of being infected due to dental practice characteristics. In Italy, from December 23, 2020, through January 2, 2021, a study was conducted to explore COVID-19 vaccine hesitancy and reasons against adherence among dentists. The researchers used an online survey with 761 dentists enlisted through the Board of Physicians and Dentists of the District of Monza Brianza in Lombardy, Italy [14]. The total number of participants was 421 dentists who completed the survey. Over 82% of participants announced their intention to adhere to the vaccination program against the COVID-19 disease. The survey questionnaire contained four answers: yes, probably yes, no, and probably no. Of the dentists participating, 59.6% answered yes, 22.6% answered probably yes, 3.8% answered no, and 14.0% answered probably no. The hesitancy rate for the COVID-19 vaccine among dentists in this study was 18% of participants. The participants' common reason for COVID-19 vaccine hesitancy was information insufficiency (39%) [14].

COVID-19 Vaccine Hesitancy Among HCWs With Booster Doses

Booster doses are defined as doses after completion of the primary vaccine series [15]. A cross-sectional survey was conducted to explore vaccine hesitancy toward the COVID-19 vaccine among US HCWs. The snowball sampling method was utilized. The total sample consisted of 1358 participants who were given the primary dose of the vaccine, and their full data was available. Of these participants, 107 (7.9%) were hesitant to take the COVID-19 vaccine, irrespective of their first or second doses [16]. The hesitancy rate toward COVID-19 was 7.9% of the participants. These 107 HCWs hesitant to receive COVID-19 vaccine doses gave their reasons as follows: 60 (56.1%) were waiting for more information on the new vaccine, 13 (12.1%) were unsure of their intention to vaccinate, and 31 (29.0%) did not plan to take the vaccine. All respondents who had taken the first dose and were hesitant about taking the second dose cited side effects with the first dose as their reason. Thus, the most common reason for hesitancy toward the COVID-19 vaccine booster doses among HCWs in the United States was side effects from the first dose [16].

Another study in Singapore was conducted as a cross-sectional study to determine the prevalence of hesitancy toward booster doses of the COVID-19 vaccine among seven primary healthcare clinic HCW staff from January 1 to December 10, 2021. This study considered the gender, profession, place of work, type, and date of the vaccine with 891 participants. On the last date of the study, December 10, 856 participants were eligible for booster doses [15]. They were divided into two groups: 558 participants (73%) were eligible and fully vaccinated, and 198 (26%) were hesitant to take booster doses. The researchers found from this study that gender, place of work, and profession do not influence factors of hesitancy. The most significant factor was that HCWs who were hesitant to take the first dose were 3.6 times more hesitant toward booster doses of the COVID-19 vaccine [15].

Racial and Ethnic Differences in COVID-19 Vaccine Hesitancy Among HCWs

Some previous studies have noted higher vaccine hesitancy among Black HCWs. In addition, no differences between Hispanic and non-Hispanic racial groups were found in this article [16].

A survey was conducted at two large academic hospitals over three weeks in November and December 2020 to investigate COVID-19 vaccine hesitancy among HCWs in various racial and ethnic groups and to consider factors related to COVID-19 vaccine hesitancy. Many studies have confirmed a difference in race and ethnicity for the severity of the COVID-19 disease. Compared to White and non-Hispanic people, Black people are two times more likely to die from COVID-19 and three times more likely to be hospitalized [17]. We found from this article that 10,871 participants, or 32.2%, completed the survey and reported their race or ethnicity. The number of individuals hesitant to accept the COVID-19 vaccine was 5540 (50% of participants). In this study, the majority of participants were White, making up 8388 (almost 77.2%). Participants included 882 Black individuals (8.1%), 845 Asian individuals (7.8%), 449 individuals of other or mixed race or ethnicity (4.1%), and 307 Hispanic or Latino participants (2.8%). Of the 882 individuals who expressed hesitancy about the COVID-19 vaccine, 732 were Black (83%), and 195 were Hispanic or Latino (63%). Both groups were more hesitant in comparison with White and Asian HCWs [17]. The main reason for COVID-19 vaccine hesitancy was fear of the side effects of the vaccine for over 87% of the participants. Other reasons for hesitancy expressed were for the newness vaccine by more than (79 %) and a lack of knowledge (75%). This study found that COVID-19 vaccine hesitancy was high among Black, Hispanic, Latino, and Asian HCWs compared with White HCWs. These results suggest a need for interventions to handle COVID-19 vaccine hesitancy among HCWs [17].

Limitations

More research is needed to determine which groups of HCW professions are more hesitant to take the COVID-19 vaccine. We need more studies to determine the accurate rate of COVID-19 vaccine hesitancy among different professions. It was difficult to confirm the exact rate of COVID-19 vaccine hesitancy among HCWs because of the limited number of studies on the various HCW professions. More research is needed to confirm these results.

## Conclusions

In this narrative review, there were varying rates of HCW hesitancy after the release of the COVID-19 vaccine. Due to the critical role of the HCWs, this issue is expected to have a significant impact on ongoing efforts to achieve widespread vaccination and herd immunity. The most common reasons are concerns about the safety and adverse effects of the vaccine, insufficient information, and a lack of confidence in healthcare policies. The recommendations to policymakers need to address these concerns by raising the awareness of PHC doctors because they are the easiest to reach and the first line for patients' information, improving communication with the HCWs through all channels (e.g., webinars, e-mails, and social media), and inviting the HCWs to online meetings or workshops with the healthcare policymakers so they can listen to and share their concerns and recommendations. It is essential to have an open and transparent evidence-based healthcare policy and to include representatives of HCWs from all professions, not just doctors, in the healthcare decision-making process. Correctly addressing the issue of HCWs' vaccination hesitancy would support efforts to contain the pandemic. In this review, although some studies have a small percentage of HCWs hesitant to receive the COVID-19 vaccine, their hesitancy may affect and impede the community from reaching herd immunity and ending the pandemic. Physicians and their colleagues should lead the world in fighting against this pandemic, be ready for any outbreak in the future, and advocate vaccination, as it is the lethal weapon against diseases. 
